# Organic modification of layered zirconium phosphate/phosphonate for controlled release of therapeutic inorganic ions

**DOI:** 10.1080/14686996.2021.1993728

**Published:** 2021-12-16

**Authors:** Jin Nakamura, Ryoya Ito, Ryohei Kozaki, Ayae Sugawara-Narutaki, Chikara Ohtsuki

**Affiliations:** a Institute for Advanced Research, Nagoya University, Nagoya, Japan; b Graduate School of Engineering, Nagoya University, Nagoya, Japan

**Keywords:** Biomaterials, layered zirconium phosphate/phosphonate, therapeutic inorganic ions, intercalation, tetrabutylammonium, 30 Bio-inspired and biomedical materials, 107 Glass and ceramic materials, 100 Materials, 301 Chemical syntheses / processing, 300 Processing / Synthesis and Recycling, 211 Scaffold / Tissue engineering / Drug delivery, 200 Applications

## Abstract

The present study aims to develop a layered zirconium phosphate/phosphonate (LZP) powder to control the release of therapeutic inorganic ions. Organically modified LZPs were successfully prepared with various contents of phenyl groups via a reflux method in an aqueous solution containing phosphoric and phenylphosphonic acids. Powder X-ray diffraction analysis and Fourier transform infrared spectroscopy revealed that the crystal structure of the synthesized LZP samples was identical to that of α-zirconium phosphate, even after modification. The amount of incorporated organic molecules increased with increasing molar fractions of phenylphosphonic acid in the starting composition, as determined from the thermal analysis. Cobalt ion (Co^2+^), a type of therapeutic inorganic ion, was incorporated into the organically modified LZP through treatment with an acetonitrile solution containing tetrabutylammonium ions, followed by treatment with an acetonitrile solution containing CoCl_2_. The amount of incorporated Co^2+^ depended on the concentration of the phenyl groups. Furthermore, the highest amount of Co^2+^ was incorporated in the sample (ZP-Ph-0.5) prepared with equimolar phosphoric/phenylphosphonic acid. The ZP-Ph-0.5 sample additionally showed the ability to incorporate copper or iron ions (Cu^2+^ or Fe^3+^). The incorporated ion, either Co^2+^ or Cu^2+^, was continuously released from the ZP-Ph-0.5 sample in a saline solution over a period of three weeks, whereas the release of Fe^3+^ was negligible. The quantity of Co^2+^ released was higher than that of Cu^2+^. The controlled release of Co^2+^ from the ZP-Ph-0.5 sample was also observed in a simulated body fluid that mimicked the ionic concentration of human blood plasma. These results confirm that a specific degree of phenyl modification makes LZP a candidate host material for releasing therapeutic inorganic ions.

## Graphical abstract

**Figure:**
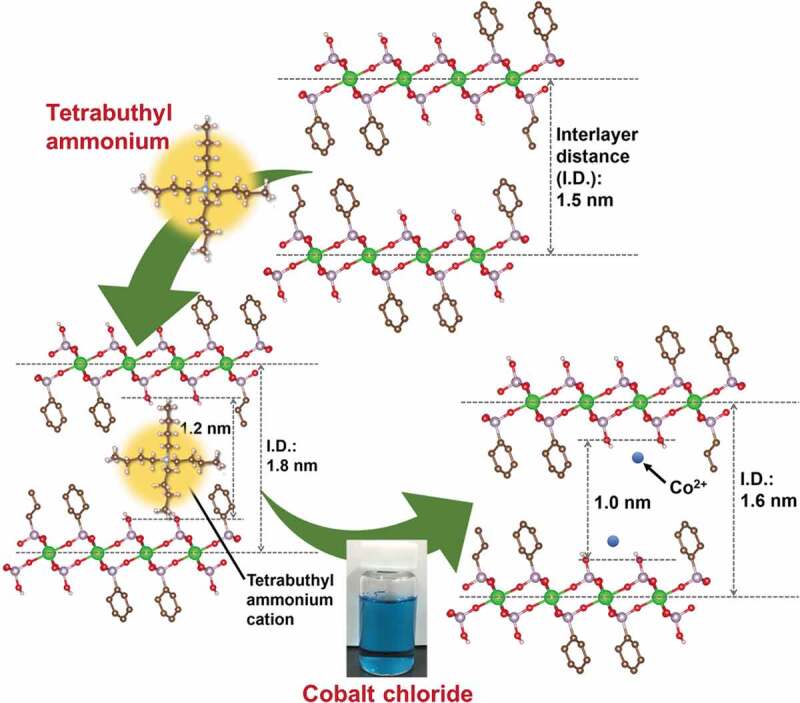

## 1. Introduction

Ceramic biomaterials are a class of materials that exhibit a specific biological affinity for biological tissues; they can be used for the reconstruction of living tissues. Bioactive ceramics have been used as materials to rectify bone defects by direct bonding with living bone after implantation at the site of defect. They have also been recognized as effective osteoconductive materials [1]. Bioactive glasses and glass-ceramics have been studied as biomaterials that show osteoconductivity after implantation at the site of defect; that is, they possess bone-bonding properties after implantation. Biomaterials with bone-bonding properties have been studied as bone substitutes. In recent years, there has been a growing demand for biomaterials that can enhance tissue regeneration and that possess antibacterial properties [2,3]. In the design of these biomaterials, controlled release of therapeutic inorganic ions and organic molecules is desired [4]. Although ceramics have been modified to incorporate organic molecules and inorganic ions as biological factors to enhance bone formation or to impart antibacterial properties, it has been challenging to achieve such physiological effects efficiently using conventional bioactive ceramic materials [5]. Therefore, the development of novel biomaterials with controlled release of therapeutic inorganic ions, as well as biologically active molecules, is necessary. As a host material for releasing biological factors, inorganic materials with layered structures have received considerable attention for controlled dissolution of the factors through the organic modification of its interlayer.

Various inorganic layered compounds, such as clays [6,7], layered double hydroxides [8,9], and layered phosphates [10,11] have been explored as biomaterials for drug delivery carriers and gene reservoirs. Among the layered compounds, the focus of this study is α-zirconium phosphate (α-ZrP: Zr(HPO_4_)_2_·H_2_O). This is because α-ZrP is made of inorganic constituents that have been intensely investigated with diverse biomedical applications, such as zirconia and phosphate. The present authors previously reported that α-ZrP and its organically modified derivatives induced adhesion of human stem cells, comparable to that of hydroxyapatite, a typical ceramic biomaterial [12]. α-ZrP is a type of layered zirconium phosphate that allows the incorporation of inorganic ions by ion exchange; furthermore, it facilitates the organic modification of the interlayers [13,14]. Thus, the organic modification of layered zirconium phosphate could lead to improved incorporation and controlled release of therapeutic inorganic ions. Although previous studies have reported the incorporation of inorganic elements into the interlayer in layered zirconium phosphate/phosphonate (LZP) [15,16], there have been few studies on the incorporation of therapeutic inorganic ions into the organically modified LZP. Furthermore, studies on its ion-releasing behavior are rare. These properties demonstrate potential for the development of substrates for novel delivery systems.

Among the therapeutic inorganic ions, cobalt ion (Co^2+^) was the focus here because of its angiogenic stimulatory effect on vascular endothelial cells [17–19]. The purpose of this study is to investigate the Co^2+^ incorporation capability of organically modified LZPs depending on the degree of modification, and to examine their ion-releasing properties. Moreover, the ability of the LZPs to incorporate copper or iron ions (Cu^2+^ or Fe^3+^) and, furthermore, their ion-release properties for these constituents were compared with those of Co^2+^. Organically modified LZP powders were prepared from the starting materials with varying amounts of phenylphosphonic acid. The present authors hypothesize that partial modification of the phosphate group with hydrophobic phenyl groups will impede the rate of ion exchange. The controlled release of Co^2+^, Cu^2+^, and Fe^3+^ from LZP will be the outcome. The incorporation of Co^2+^ into each organically modified LZP powder was then attempted using tetrabutylammonium (TBA)-modified LZP. Furthermore, the release behavior of the incorporated Co^2+^, Cu^2+^, and Fe^3+^ ions from each organically modified LZP was investigated in a saline solution.

## 2. Experiments

### 2.1. Preparation of organically modified LZP

For the synthesis of α-ZrP and its derivatives under suitable conditions with appropriate modifications, the preparation procedures were according to a previous report [15]. All chemicals were used as received without further purification. Zirconyl chloride octahydrate (5.0 g; ZrOCl_2_ · 8H_2_O, Kanto Chemical, Japan), and appropriate amounts of phosphoric acid solution (85%; H_3_PO_4_, Nacalai Tesque, Japan) and phenylphosphonic acid (C_6_H_5_PO_3_H, Tokyo Chemical Industry, Japan), were added to 48 mL of deionized water. The P/Zr molar ratio of the starting material is two. The mixture was refluxed at 100°C for 24 h with stirring. The product was centrifuged at 6500 × *g* for 10 min (MX-301, TOMY SEIKO, Japan) and upon discarding the supernatant, dispersed in deionized water (40 mL) through sonication. These clean-up steps were repeated three times. Subsequently, the sample was dried for 1 d at 65°C in a drying oven (DRYING OVEN DO-300, IUCHI, Japan), followed by pulverization. Hereafter, the samples are denoted as ZP-Ph-*x*, where *x* equals 0, 0.25, 0.50, 0.75, or 1.0, as given in Table 1.

**Table 1.** Prepared samples with different amounts of phenyl group

Sample code	Molar ratio of phenylphosphonate: phosphoric acid in starting composition
ZP-Ph-0	0: 100
ZP-Ph-0.25	25: 75
ZP-Ph-0.5	50: 50
ZP-Ph-0.75	75: 25
ZP-Ph-1.0	100: 0

Powder X-ray diffraction (XRD) patterns of the ZP-Ph-*x* samples were obtained using Cu Kα radiation on an X-ray diffractometer (RINT-2100/PC, Rigaku, Japan) at scanning rates of 1 and 0.5°/min for measurement ranges 2 *θ *= 2–40° and 2–10°, respectively, to observe in detail the changes in the interlayer distance (ID). The samples were also characterized by differential thermal and thermogravimetric analysis (TG-DTA, DTG-60, Shimadzu, Japan) as well as Fourier transform infrared spectroscopy (FTIR, FT/IR-6100, Jasco, Japan) using the KBr pellet method. The temperatures at the onset and end of mass loss were estimated by extrapolation of the thermogravimetric (TG) curves (denoted hereafter as the extrapolation method) unless specifically mentioned. The temperatures were estimated from the intersecting points between a tangential line drawn to the steepest point of the mass loss and the extrapolated baseline before and after mass loss. The morphologies of the samples were observed using a transmission electron microscope (TEM, JEM-2100Plus, JEOL, Japan). The specific surface area of the ZP-Ph-x particles was measured using the Brunauer Emmett Teller (BET) nitrogen adsorption method (Nova 1000e, Quantachrome Instruments, USA).

### 2.2. Incorporation of cobalt, copper, and iron into ZP-Ph-x samples and evaluation of their releasing behavior

The incorporation of cobalt into the samples was carried out through the subsequent addition of an acetonitrile solution containing CoCl_2_ to the ZP-Ph-*x* powder treated with an acetonitrile solution containing TBA. Specifically, after 0.2 g of ZP-Ph-*x* was dispersed in a solution (TBA solution) of acetonitrile (CH_3_CN, Fujifilm Wako Pure Chemical, Japan) and tetrabutylammonium hydroxide (40%, (C_4_H_9_)_4_NOH, Tokyo Chemical Industry, Japan) at a volume ratio of 19.5:0.5 (mL), the slurry was kept at approximately 25°C. After standing for 2 d, the powder was collected by centrifugation at 7300 × *g* for 10 min and characterized by powder XRD.

To achieve the incorporation of Co^2+^ into the ZP-Ph-*x* samples, a CoCl_2_ solution was prepared by dissolving cobalt (II) chloride hexahydrate (7.1 mg) (CoCl_2_ · 6H_2_O, Nacalai Tesque, Japan) in acetonitrile (20 mL). This resulted in the concentration of Co^2+^ in the solution being 1.5 mmol·L^−1^, which was subsequently added to the centrifuged samples after treatment with the TBA solution. Specifically, the sample were dispersed in the aforementioned CoCl_2_ solution by shaking at approximately 25°C using a shaking bath (LT-10 F, Taitec, Japan). After shaking for 1 d, the sample powders were collected by centrifugation at 7300 x *g* for 10 min. The precipitates were analyzed using powder XRD, whereas the concentration of residual Co^2+^ in the supernatant was determined using inductively coupled plasma atomic emission spectroscopy (ICP-AES, Optima 2000 DV, PerkinElmer, USA). The samples for the evaluation of yield after treatment with TBA solution followed by centrifugation were separately prepared following the described procedures and then dried at 40°C for 3 d.

The incorporation of Cu^2+^ or Fe^3+^ was monitored using ultraviolet–visible (UV–vis, V-670, Jasco, Japan) absorption spectrometry. Copper acetate (II) monohydrate ((CH_3_COO)_2_Cu·H_2_O, Fujifilm Wako Pure Chemical, Japan) and iron (III) chloride hexahydrate (FeCl_3_·H_2_O, Kishida Chemical, Japan) were used as starting materials for the incorporation of Cu^2+^ and Fe^3+^, respectively, into ZP-Ph-0.5, which showed a high capability of incorporating Co^2+^. The incorporated amounts of Cu^2+^ and Fe^3+^ were estimated from the concentration of residual Cu^2+^ and Fe^3+^ in the supernatant, as determined from the absorbance at 252 and 370 nm for Cu^2+^ and Fe^3+^, respectively, in the UV–vis absorption spectra.

The release behavior of Co^2+^, Cu^2+^, and Fe^3+^ from ZP-Ph-0.5 incorporated with these ions was evaluated in a saline solution with a NaCl concentration of 0.9 wt%. Each sample powder (20 mg) was immersed in a saline solution (10 mL) and placed in an incubator (MIR-162, SANYO, Japan) at 37°C for a certain period (2, 4, 6, 8, 24, 72, and 168 h). The powders were collected through centrifugation at 7300 × *g* for 10 min and dispersed in a renewed 0.9 wt% NaCl solution (10 mL) through sonication, whereas the concentrations of Co^2+^, Cu^2+^, and Fe^3+^ in the supernatant were determined using ICP-AES. The release behavior was monitored through changes in the concentration of ions in the supernatants obtained after each immersion period (2, 4, 6, 8, 24, 72, and 168 h).

The release behavior of Co^2+^ from the ion-incorporated ZP-Ph-0.5 was also investigated at 37°C in both a simulated body fluid (SBF, pH 7.4) and water using a procedure similar to that for saline. To prepare the SBF, ultrapure water (700 mL) was added to a beaker. The reagents listed in Table S1 were then dissolved in the order listed under stirring. Each reagent was completely dissolved before the addition of the following reagents. The pH of the solution was adjusted to 7.4 by adding 1.0 mol·L^−1^ hydrochloric acid at 36.5°C; the solution was then transferred to a volumetric flask and ultrapure water was added to adjust the total volume to 1 L. The SBF or water was renewed at immersion periods of 2, 4, 6, 8 and 24 h.

0.05 g of ZP-Ph-0.5 powder incorporated with Co^2+^ was dispersed in 1 mL of ethanol via sonication. A sub-sample of the obtained dispersion (50 µL) was spread on one side of a polyethylene disc (PE disc, diameter 14 mm, PEN-050501, AS ONE Co., Japan) preheated at 90°C on a hot plate (HHP-140D, AS ONE Co., Japan). The amount of sample powder on a PE disk was adjusted to be 2.5 mg. The substrate was then hot-pressed using hotplate and stainless-steel weight blocks (350 MPa for 30 s at 140°C). The prepared substrate was placed in each well of a 24-well plate (Cellstar 662,160, Greiner Bio-One International, Austria) with the powder-embedded side facing upward. Subsequently, 1.25 mL of the SBF or saline was added to each well. The 24-plate was placed in an incubator (EL-600 V, AS ONE Co., Japan) for 24 h at 37°C, and the concentrations of Co^2+^ in the supernatants were measured using ICP-AES.

## 3. Results

### 3.1. Characterization of the prepared ZP-Ph-x powders

**Figure 1.**
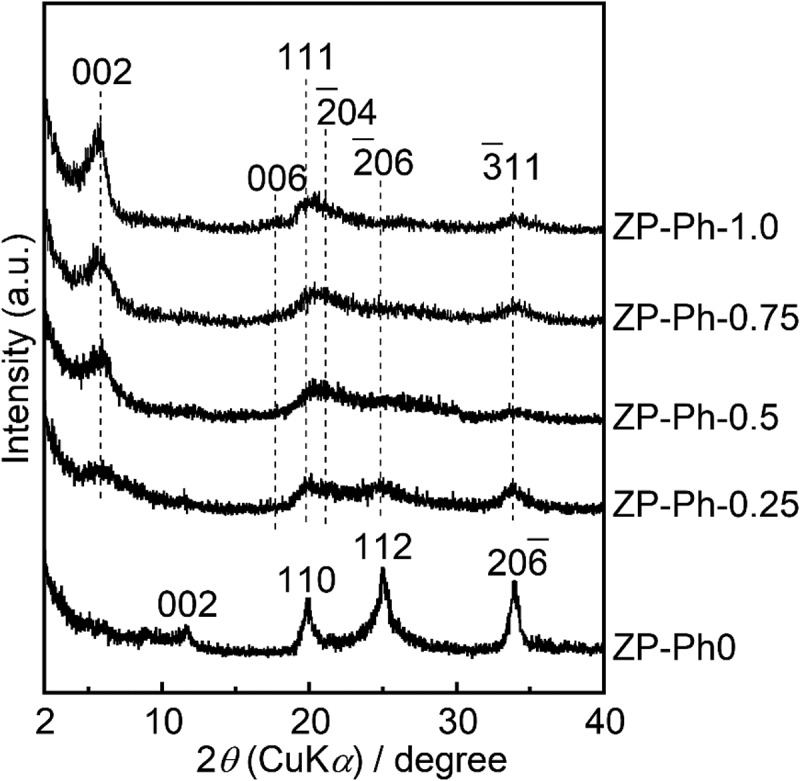
Powder XRD patterns of the ZP-Ph-*x* samples. Spectra are vertically offset for presentation purposes

Figure 1 shows the powder XRD patterns of the prepared ZP-Ph-*x* samples. The diffraction pattern of ZP-Ph-0, which is free from organic modification, shows 002, 110, 112, and $\overset{ˉ}{2}$06 reflection peaks that were assigned to α-ZrP according to the International Centre for Diffraction Data card ICDD 33–1482. The diffraction patterns of the sample powders subjected to organic modification, that is, *x* = 0.25, 0.50, 0.75, and 1.0, were assigned according to ICDD 44–2000. The sample powders subjected to organic modification showed a 002 reflection shift from 2*θ* = 11° to approximately 2*θ* = 6°. Broad reflection peaks were observed in the range 2*θ* of 20–40°, corresponding to the 111, $\overset{ˉ}{2}$04, $\overset{ˉ}{2}$06, and $\overset{ˉ}{3}$11 reflections of zirconium phenylphosphonate. The lowered shift of the 002 diffraction peak was caused by the increase in ID with organic modification (the incorporation of phenyl groups), even in the ZP-Ph-0.25 sample. The higher concentration of phenyl groups in the organic modification process led to a higher intensity of 002 reflection even after organic modification, as can be seen in the diffraction patterns of ZP-Ph-0.75 and ZP-Ph-1.0. The estimated ID in ZP-Ph-0 (α-ZrP) was 0.8 nm, whereas that in ZP-Ph-1.0 was 1.6 nm. Disordered crystalline structures were observed for ZP-Ph-0.25 and ZP-Ph-0.5.

**Figure 2.**
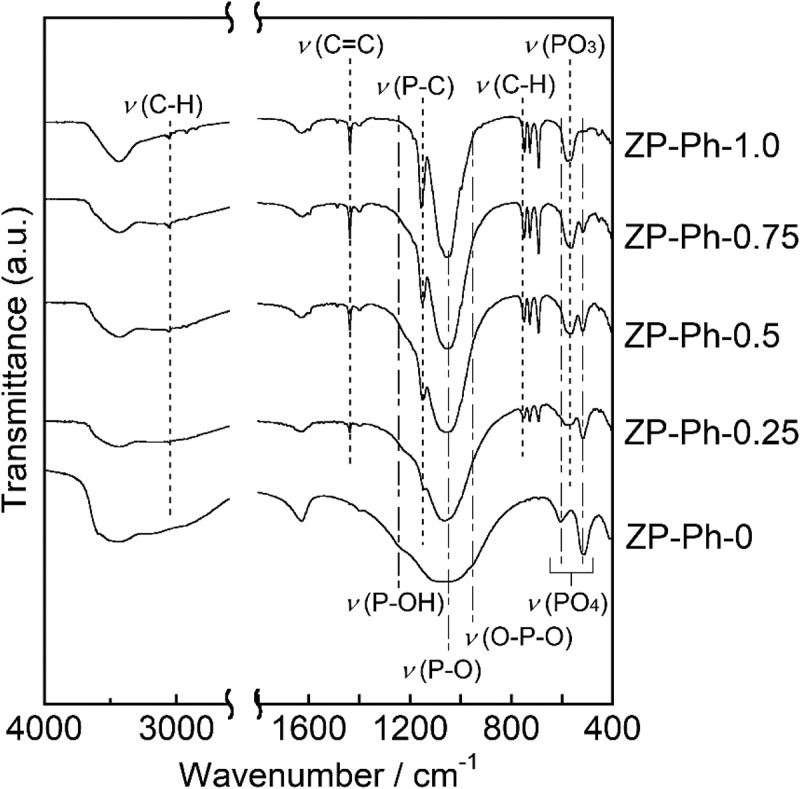
FTIR spectra of the ZP-Ph-*x* samples. Spectra are vertically offset for presentation purposes

Figure 2 shows the FTIR spectra of the prepared ZP-Ph-*x* samples. ZP-Ph-0 showed broad absorption peaks with wavenumbers between 950 and 1100 cm^−1^, which were assigned to P-O vibrations. The absorption peak centered at 1050 cm^−1^ was assigned to the P-O symmetric stretching vibration, whereas that at 960 cm^−1^ corresponds to the O-P-O bending vibration. The organically modified samples ZP-Ph-0.25, ZP-Ph-0.5, ZP-Ph-0.75, and ZP-Ph-1.0 showed absorption peaks at 750, 1156, and 3061 cm^−1^, which were assigned to C-H, P-C, and C-H bonds in the phenylphosphonic component, respectively. The absorption intensities for these peaks increased with increasing amounts of phenylphosphonic acids in the starting composition in the following order: ZP-Ph-0.25 < ZP-Ph-0.5 < ZP-Ph-0.75 < ZP-Ph-1.0. Conversely, the intensity of the absorption peak at 1245 cm^−1^, which was assigned to P-OH bonds, decreased with increasing amounts of phenylphosphonic acid in the starting compositions. The peaks at 1450 cm^−1^ correspond to the stretching of the C = C bonds in the phenyl group. The peak at 570 cm^−1^ was assigned to the bending vibration of the PO_3_ group. The peaks at 610 and 520 cm^−1^ were assigned to the deformation of the PO_4_ group. Among the ZP-Ph-X samples, ZP-Ph-1.0 had the lowest relative intensity of the peak at 520 cm^−1^. This suggests that the quantity of PO_4_ groups in ZP-Ph-1.0 was minimized corresponding to the preparation composition.

**Figure 3.**
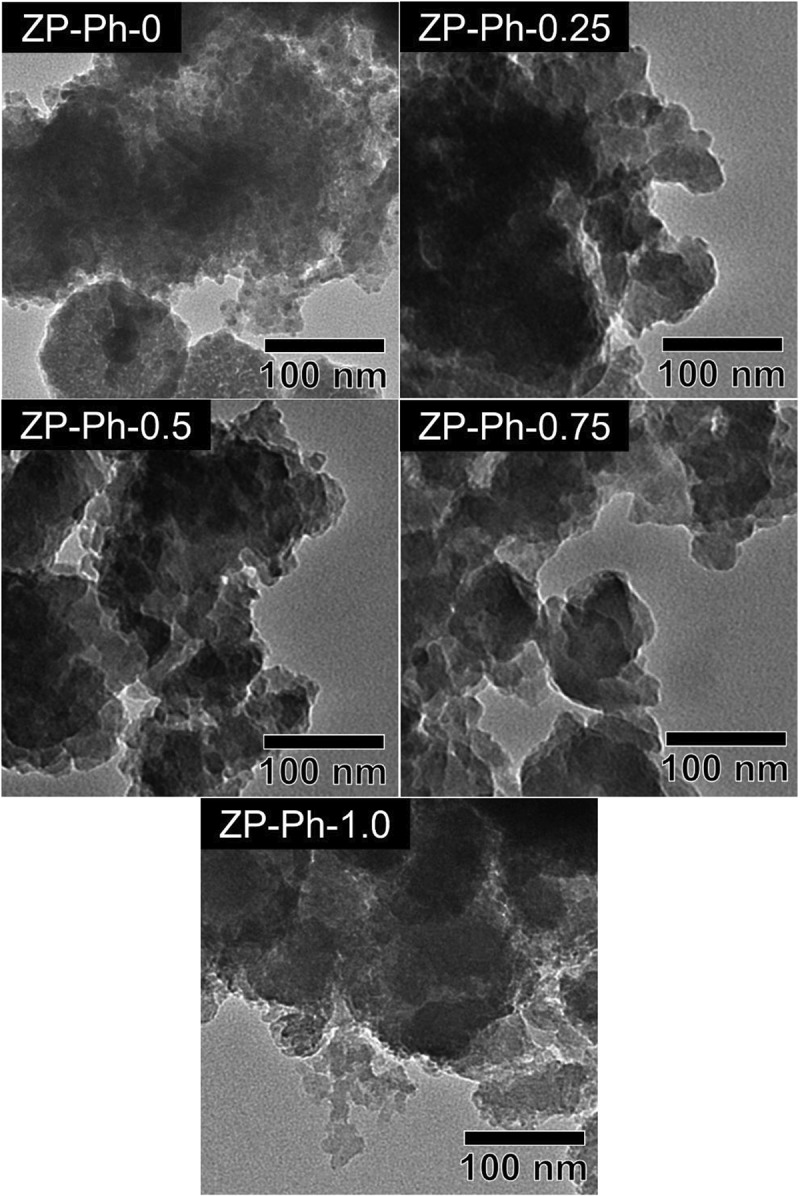
TEM images of the ZP-Ph-*x* samples

**Figure 4.**
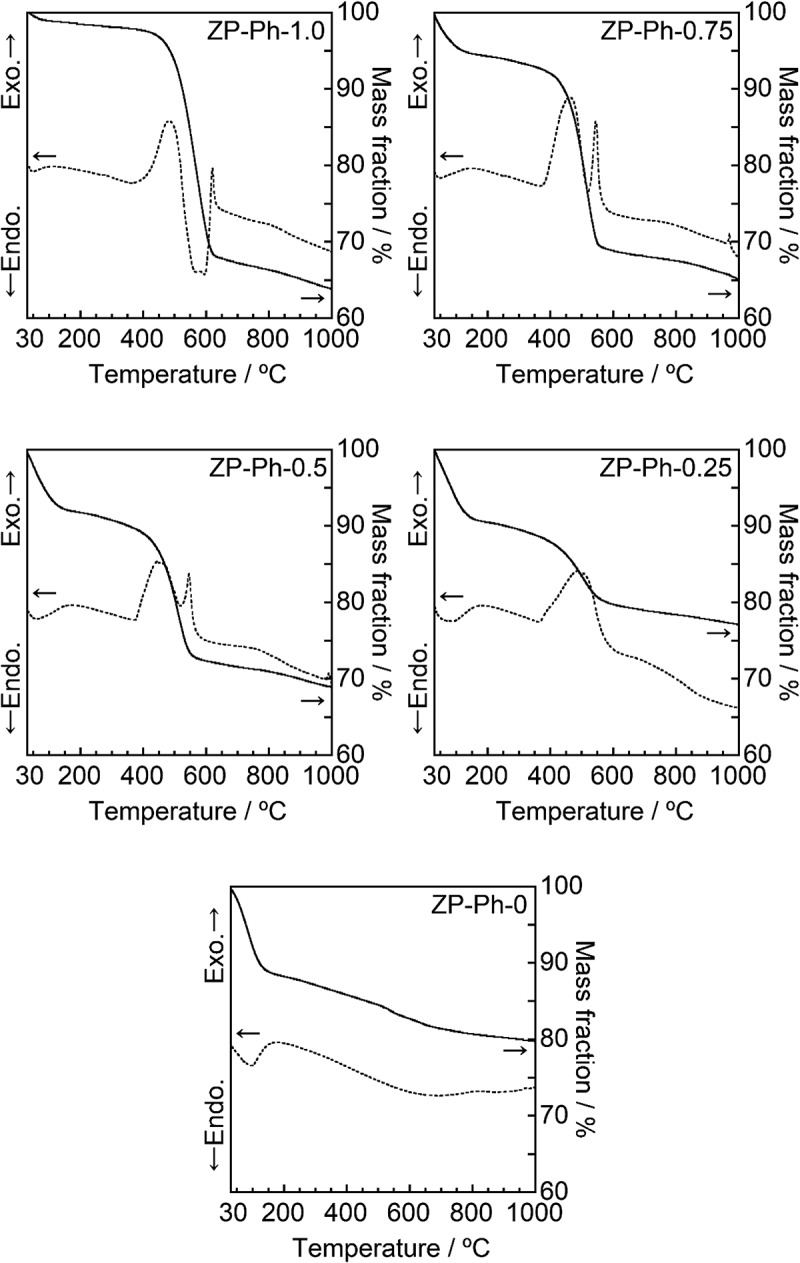
TG-DTA curves of the ZP-Ph-*x* samples

Figure 3 shows the TEM images of the prepared ZP-Ph-*x* samples. All prepared ZP-Ph-*x* samples (*x* = 0, 0.25, 0.50, 0.75, and 1.0) showed similar morphologies, that is, the aggregates of the particles had diameters of ~10 nm. The specific surface area (SSA) of these samples was measured as 21, 103, 105, 155 and 146 m^2^/g for ZP-Ph-0, ZP-Ph-0.25, ZP-Ph-0.5, ZP-Ph-0.75, and ZP-Ph-1.0, respectively. Figure 4 shows the TG (solid line) and differential thermal analysis (DTA, dashed line) curves of the prepared ZP-Ph-*x* samples. ZP-Ph-0 showed a significant mass loss below 150°C, accompanied by an endothermic peak, which was attributed to elimination of water from the interlayers of the α-ZrP crystal [20]. Thus, the mass losses of the prepared samples below 150°C were ascribed to the removal of water. ZP-Ph-0 showed a gradual mass loss with a broad endothermic peak at 500–800°C, which could be ascribed to the dehydration of -OH groups at the interlayer to form pyrophosphate (P-O-P) groups. The TG and DTA curves of the samples prepared with phenylphosphonic acid also showed notable mass loss between 420 and 650°C accompanied by exothermic peaks, which were attributed to the combustion of the organic components of phenyl-modified zirconium phosphate/phosphonate under ambient conditions. Based on the TG results, the mass losses caused by the removal of water and combustion of organic components were estimated using the extrapolation method; the results are summarized in Table 2. The initial temperature of the TG analysis (35°C) was considered as the onset temperature for the removal of water because it was difficult to find the baseline for the method. When the fraction of phenylphosphonic acid in the starting composition increased, the fraction of the lost water mass decreased, whereas conversely, the fraction of the combusted organic components increased.

**Table 2.** Estimated mass losses corresponding to water molecules and organic functional groups in the samples. The estimated temperature ranges were determined by the extrapolation method

Sample code	Interlayer water molecules		Organic functional groups	
	Estimated temperature range/°C	Mass loss / %	Estimated temperature range/°C	Mass loss / %
ZP-Ph-0	45–127	11	No data	0
ZP-Ph-0.25	38–126	9	425–557	8
ZP-Ph-0.5	30–110	8	444–544	16
ZP-Ph-0.75	31–97	5	455–544	23
ZP-Ph-1.0	No data	0	502–613	30

### 3.2. Incorporation of cobalt into ZP-Ph-x samples

**Figure 5.**
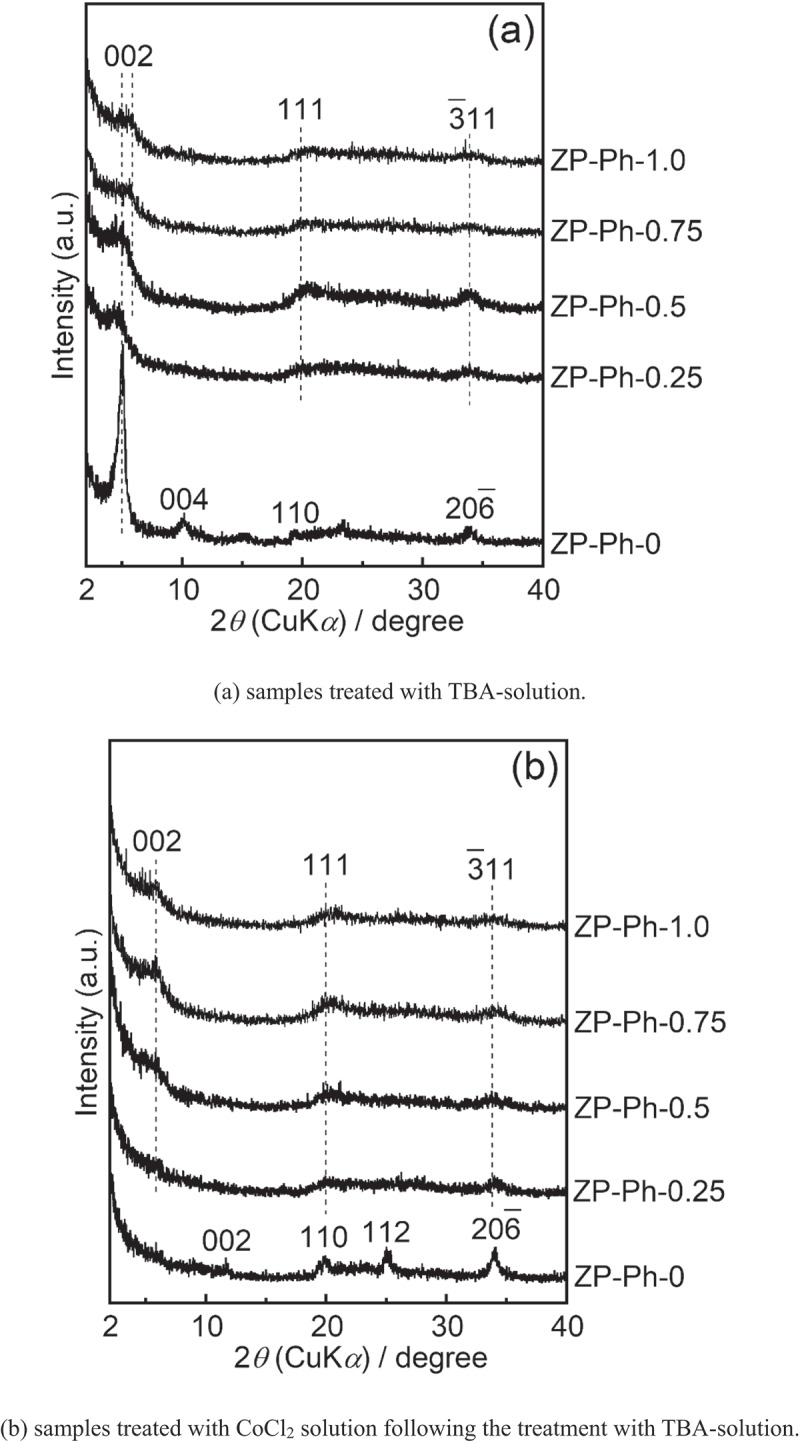
Powder XRD patterns of the samples soaked in acetonitrile solution containing TBA (TBA-solution) for 2 d, as well as those of the samples treated with the TBA-solution followed by CoCl_2_ solution (CoCl_2_ solution). (a) samples treated with TBA-solution. (b) samples treated with TBA-solution followed by treatment with CoCl_2_ solution. Spectra are vertically offset for presentation purposes

Figure 5 shows the powder XRD patterns of the samples immersed in an acetonitrile solution containing TBA (TBA solution) for 2 d (Figure 5(a)), as well as those of the samples subsequently treated with the CoCl_2_ solution following treatment with the TBA solution (Figure 5(b)). For the ZP-Ph-0 sample, the reflection peaks appeared at 2*θ* = 5.1° and 10.1° (d = 1.8 and 0.9 nm, respectively); these peaks were attributed to the 002 and 004 reflections of α-ZrP after intercalation of the TBA molecule [21]. A decrease in intensity of the peak at 2*θ* = 11.7°, which was assigned to the 002 reflection of α-ZrP, was observed. The other peaks of ZP-Ph-0 treated with TBA solution showed a slight decrease in intensity without any shifts compared to those of the as-prepared ZP-Ph-0. The pattern of ZP-Ph-0 subsequently treated with CoCl_2_ showed a decrease in intensity of the peak attributed to the 002 and 004 reflections. The intensity of peaks attributed to the 002 reflections in ZP-Ph-0.25, ZP-Ph-0.5, ZP-Ph-0.75, and ZP-Ph-1.0 decreased upon treatment with TBA solution. These reductions are considered to originate from the swelling of the interlayer due to the treatment. After treatment with CoCl_2_, the ZP-Ph-0, ZP-Ph-0.25, ZP-Ph-0.50, ZP-Ph-0.75, and ZP-Ph-1.0 samples showed no significant decrease in the intensities of the 002, 110, 112, and $\overset{ˉ}{2}$06 peaks.

**Figure 6.**
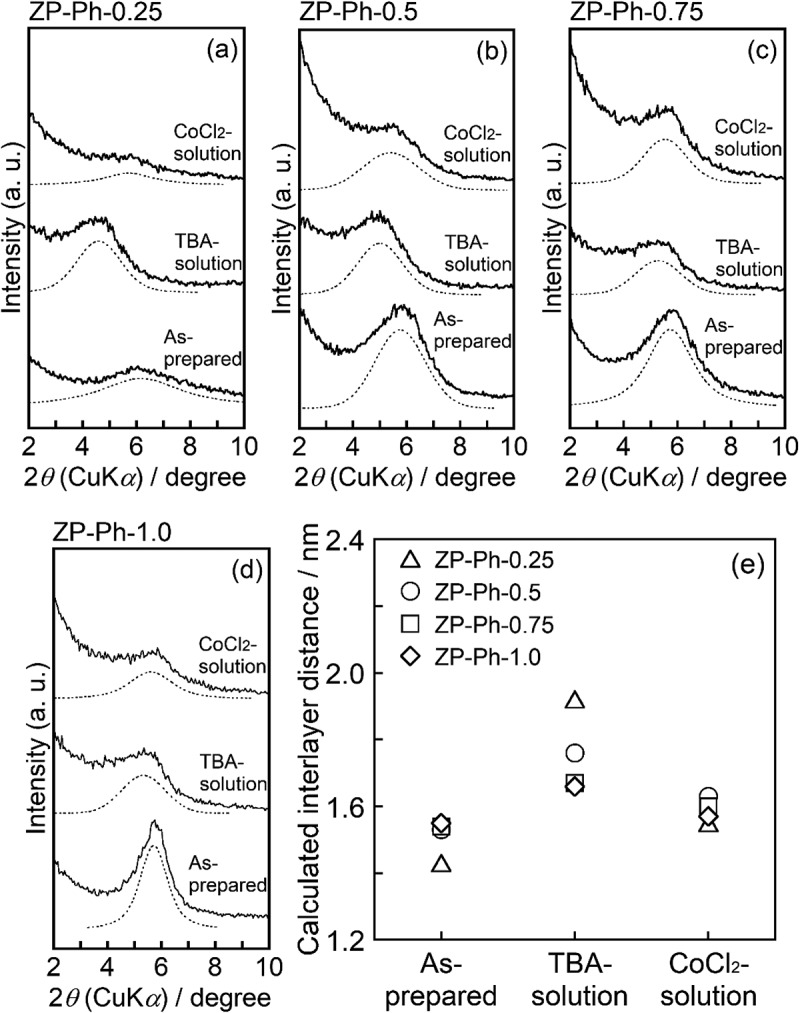
Powder XRD patterns of the as-prepared samples (as-prepared), the samples with the TBA-treatment (TBA-solution), and the samples treated with TBA-solution followed by CoCl_2_ solution (CoCl_2_ solution). (a) ZP-Ph-0.25, (b) ZP-Ph-0.5, (c) ZP-Ph-0.75, (d) ZP-Ph-1.0 and (e) summary of calculated interlayer distances. Spectra are vertically offset for presentation purposes

For a detailed discussion on the changes in the ID of ZP-Ph-*x* (*x* = 0.25–1.0), slowly scanned profiles ranging from 2*θ* = 2° to 10° were investigated (Figure 6). The powder XRD patterns of the as-prepared samples, samples treated with TBA solution, and samples treated with CoCl_2_ solution were compared. In the XRD profile of ZP-Ph-0.25, a broad diffraction peak at 2*θ* = 6.2° was observed. Another broad peak at approximately 2*θ* = 4.6° appeared after the treatment with TBA solution. Moreover, the ZP-Ph-0.25 sample treated with CoCl_2_ showed a broad peak at approximately 2*θ* = 5.7°. For ZP-Ph-0.5, a broad peak appeared at 2*θ* = 5.8°, and another broad peak was observed at approximately 2*θ* = 5.4° after CoCl_2_ treatment. The peak appearing at 2*θ* = 5.0° was assigned to the enlargement of ID after treatment with TBA solution. An increase in the intensity of the peak at approximately 2*θ *= 6° was observed with an increase in the quantity of organic groups in the interlayer in as-prepared samples. The appearance of broad peaks at approximately 2*θ* = 5° on ZP-Ph-0.25, ZP-Ph-0.5, and ZP-Ph-0.75 after treatment with TBA solution indicated that TBA was intercalated in the interlayer. However, the ID of the ZP-Ph-0.25 and ZP-Ph-0.5 samples treated with CoCl_2_ exhibited diffraction angles similar to those of the as-prepared samples. Fig. S1 shows the FTIR spectra of ZP-Ph-0.5 after treatment with TBA solution and after TBA treatment followed by CoCl_2_. The sample treated with TBA solution showed absorption peaks at 1382, 1462, and 1486 cm^−1^, which were assigned to the C-H bond in the TBA molecules. These peaks were absent for the CoCl_2_ treated sample indicating that partial organic modification with phenyl groups allowed the intercalation of TBA molecules, and TBA was de-intercalated after treatment with CoCl_2_. ZP-Ph-1.0 showed a small peak shift after treatment with TBA solution, along with an increase in the peak width. This result suggests that the organically modified layer was strongly stacked during the treatment with TBA. The masses of the dried powder samples after treatment with TBA followed by centrifugation were measured. The masses were 0.20, 0.09, 0.06, 0.05 and 0.15 g for ZP-Ph-0, ZP-Ph-0.25, ZP-Ph-0.5, ZP-Ph-0.75 and ZP-Ph-1.0, respectively.

**Figure 7.**
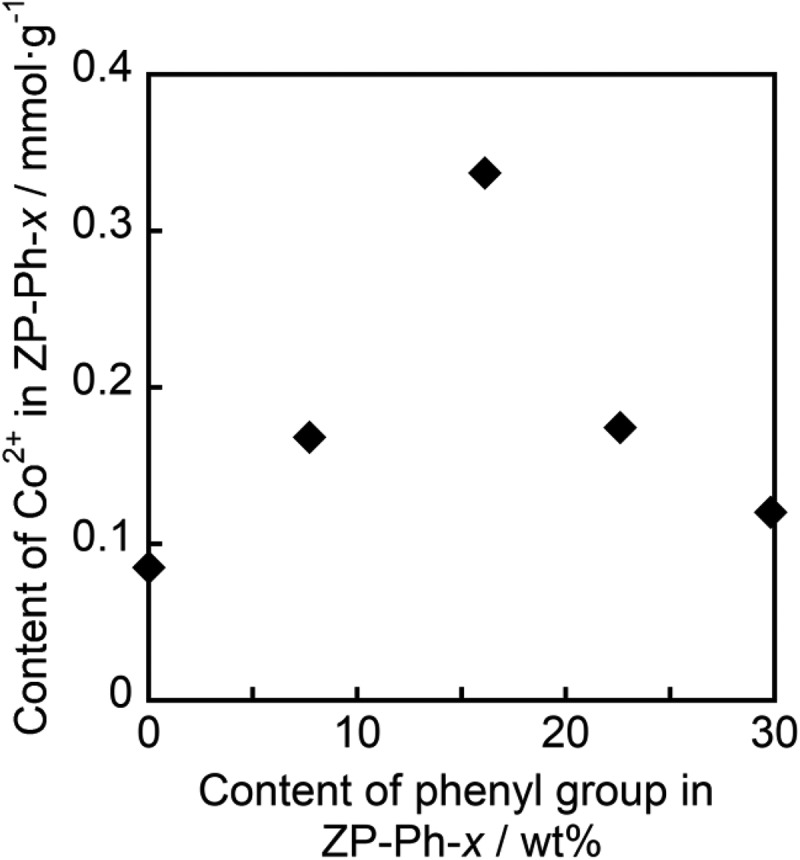
Relationship between estimated contents of Co^2+^ and phenyl groups in the sample powders

The supernatants from the CoCl_2_ treatment step were analyzed using ICP-AES to determine the Co^2+^ content in the samples. Figure 7 shows the relationship between the contents of Co^2+^ and phenyl groups in the organically modified LZPs, as estimated from the TG curves of the samples. Maximum Co^2+^ content was observed in ZP-Ph-0.5: 0.3 mmol·g^−1^. Therefore, ZP-Ph-0.5 was chosen as the sample for loading ions and for evaluating the ion-releasing behavior.

### 3.3. Release of inorganic ions from ZP-Ph-0.5

**Figure 8.**
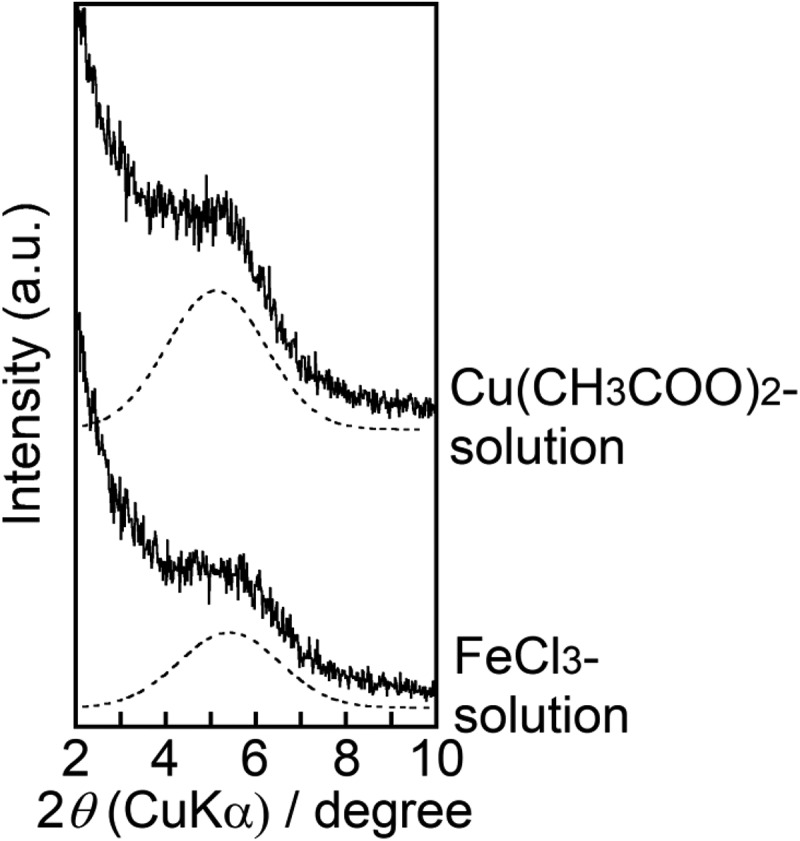
Powder XRD patterns of the ZP-Ph-0.5 treated with TBA-solution followed by Cu(CH_3_COO)_2_ solution or FeCl_3_ solution

Cu^2+^ and Fe^3+^ were incorporated into ZP-Ph-0.5 by immersing the sample in acetonitrile solutions containing either copper acetate (Cu(CH_3_COO)_2_) or iron chloride (FeCl_3_), respectively, following TBA treatment. Figure 8 shows the XRD patterns of the obtained samples. The samples treated with the Cu(CH_3_COO)_2_ or FeCl_3_ solutions showed peaks at 2*θ* = 5.1 or 5.4° (d = 1.7 or 1.6 nm), respectively. These diffraction angles were close to those of ZP-Ph-0.5 processed with CoCl_2_ (Figure 5). Moreover, the ID in this case was shorter than that after treatment with TBA (2*θ* = 5.0°, d = 1.8 nm). Therefore, the introduction of Cu^2+^ and Fe^3+^ in the interlayer could have occurred. After measuring the residual ion concentrations in the solution by UV–vis spectrometry, the determined amounts of Cu^2+^ and Fe^3+^ incorporated into 1 g of ZP-Ph-0.5 were 1.0 and 0.5 mmol·g^−1^, respectively.

**Figure 9.**
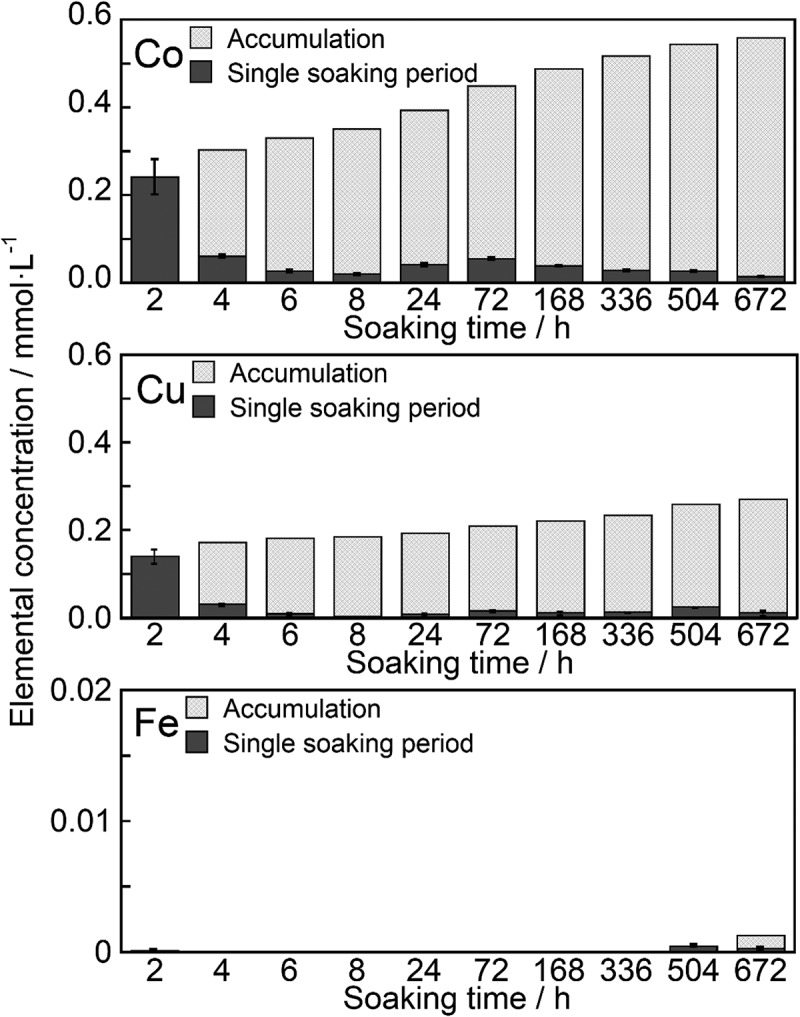
Release behaviors of ions from the ZP-Ph-0.5 which was treated with TBA-solution followed by CoCl_2_ solution (Co), Cu(CH_3_COO)_2_ solution (Cu) or FeCl_3_ solution (Fe)

The samples incorporated with Co^2+^, Cu^2+^, and Fe^3+^ were immersed in a saline solution that was changed at certain specific periods. Figure 9 shows the Co^2+^, Cu^2+^, and Fe^3+^ concentrations of the saline after sample immersion. The filled and meshed bars indicate the concentrations for the individual immersion period and accumulated concentrations, respectively. Co^2+^ showed rapid dissolution within 2 h of immersion. The concentration of Co^2+^ reached 0.242 mmol·L^−1^. Sustained release of Co^2+^ was observed from 4 to 672 h after immersion. The release rate was in the range 0.1–0.5 µmol·L^−1^·h^−1^ after 24 h of immersion. Similarly, the sustained release of Cu^2+^ was observed following rapid dissolution for up to 2 h after immersion. The releasing rate of Cu^2+^ was approximately 2 µmol·L^−1^·h^−1^ between 24 and 72 h after immersion, which then decreased to 0.1–0.5 µmol·L^−1^·h^−1^. The release of Fe^3+^ from the samples was negligible.

**Figure 10.**
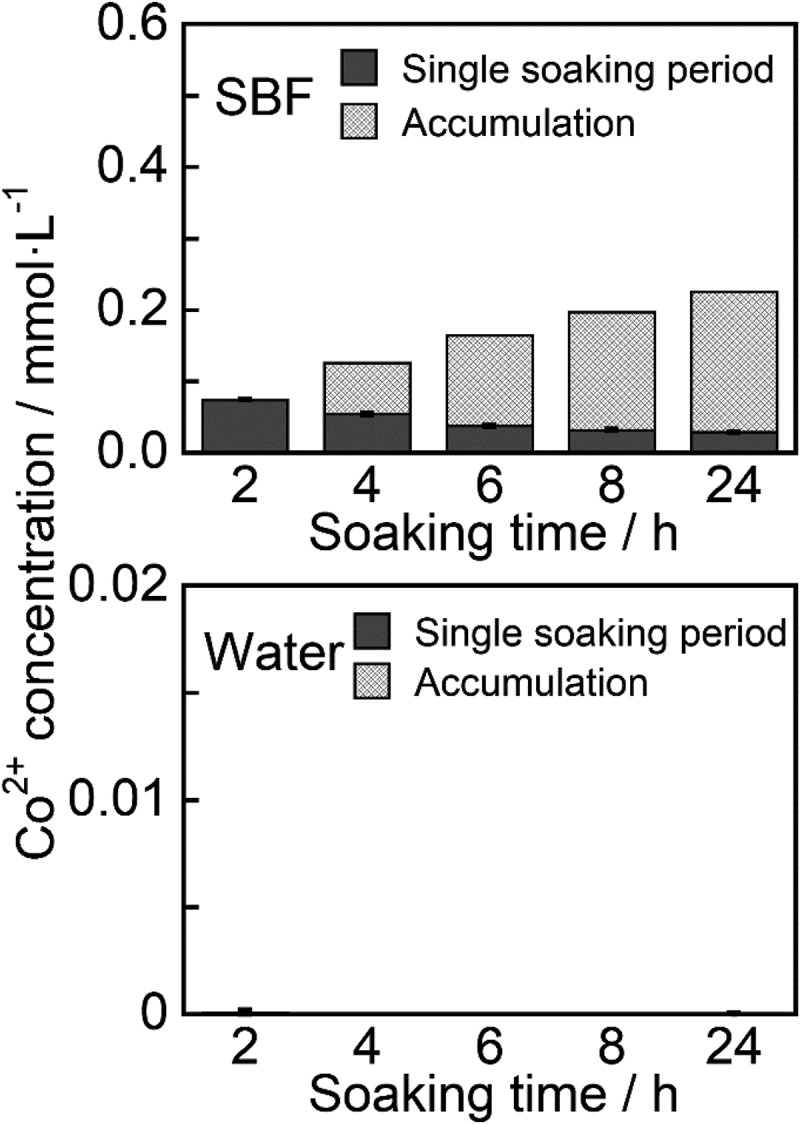
Release behaviors of Co^2+^ from the ZP-Ph-0.5 into simulated body fluid (SBF) and water

The release behavior of Co^2+^ from ZP-Ph-0.5 was also evaluated by immersion in SBF and water for 24 h. Figure 10 shows the concentrations of Co^2+^ in SBF and water after immersion of the samples. The concentration reached 0.074 mmol·L^−1^ within the initial 2 h. Sustained release of Co^2+^ was observed from 2 h to 24 h after immersion. The release rate was in the range 2–37 µmol·L^−1^·h^−1^. The release of Co^2+^ from water was negligible throughout the experimental period.

To investigate the effect of polymer embedding on ion release, Co^2+^-incorporated ZP-Ph-0.5 powder was hot-pressed onto one side of the PE substrate, and then immersed in SBF or saline. The Co^2+^ concentrations in the SBF and saline reached 0.062 and 0.125 mmol·L^−1^, respectively, after immersion of the substrates for 24 h.

## 4. Discussion

Layered materials have structures wherein heterolayers with a thickness of a few atomic layers are stacked via weak interactions, such as electrostatic forces with counter-charged ions or hydrogen bonding with water molecules. Because of the structural flexibility at the interlayer, organic drug molecules and inorganic ions of various sizes can be inserted between the layers on the basis of ion-exchange chemistry. Organically modified LZPs have been explored as excellent host materials to incorporate guest organic molecules via intercalation [22–24] or ion exchange [15,25,26]. The availability of acidic groups such as phosphate and sulfonate (SO_3_H) groups in the organically modified LZP is key for the uptake of inorganic ions caused by the ion-exchange property. Organically modified LZP with available acidic groups has been prepared using a mixture of phosphoric and aromatic phosphonic acids as the starting materials [15] and through post-synthesis sulfonation of the aromatic groups [26]. Here, phenyl-modified zirconium phosphate/phosphonate was prepared using a mixture of phosphoric and phenylphosphonic acids at various molar fractions.

From the XRD results, the ID calculated from the peak of the 002 reflection was 1.48 nm in the sample synthesized using phenylphosphonic acid as the starting material (ZP-Ph-1.0). This ID was identical to the distance of zirconium phenylphosphonate (Zr(C_6_H_5_PO_3_)_2_) reported by Alberti et al. [27]. Furthermore, broad peaks observed at 2*θ* = 19.5°, 21.1°, 24.8°, and 33.5° were known to originate from 111, $\overset{ˉ}{2}$04, $\overset{ˉ}{2}$06, and $\overset{ˉ}{3}$11 of zirconium phenylphosphonate (ICDD 44–2000), respectively, as reported by Poojary et al. [28]. These results indicate that ZP-Ph-1.0 has a structure in which a layered skeleton of zirconium phosphate/phosphonate is laminated by a phenyl group. ZP-Ph-0.75, ZP-Ph-0.5, and ZP-Ph-0.25 showed diffraction peaks at angles identical to those of ZP-Ph-1.0. The presence of phenyl groups was also confirmed by the presence of *ν*(P-C) and *ν*(C-H) peaks in the FTIR spectrum. These results collectively indicate the successful formation of phenyl-modified LZPs at various molar fractions of phenylphosphate/phosphoric acid in the starting materials. Table 3 lists the compositions of the samples estimated from the TG-DTA data in Table 2. The estimated composition of the C_6_H_5_PO_3_ group in the samples decreased to 60%–70% of the theoretical composition estimated from the compositions of the starting materials. Moreover, the FTIR spectra of the samples showed a broad peak at approximately 3500 cm^−1^, which corresponds to the *ν*(O-H) of the phosphate groups. These results indicate that the content of phenyl groups in the prepared samples increased with increasing molar fraction of the starting materials. In addition, some of the phenyl groups underwent hydrolysis to form phosphate (PO-H) groups during sample preparation.

**Table 3.** Compositions estimated from mass fractions corresponding to water molecules and organic functional groups in the samples listed in Table 2

Sample code	Estimated composition
ZP-Ph-0	Zr(HPO_4_)_2_ · 2.0H_2_O
ZP-Ph-0.25	Zr(C_6_H_5_PO_3_)_0.3_(HPO_4_)_1.7_ · 1.6H_2_O
ZP-Ph-0.5	Zr(C_6_H_5_PO_3_)_0.7_(HPO_4_)_1.3_ · 1.5H_2_O
ZP-Ph-0.75	Zr(C_6_H_5_PO_3_)_1.1_(HPO_4_)_0.9_ · 1.0H_2_O
ZP-Ph-1.0	Zr(C_6_H_5_PO_3_)_1.4_(HPO_4_)_0.6_

**Figure 11.**
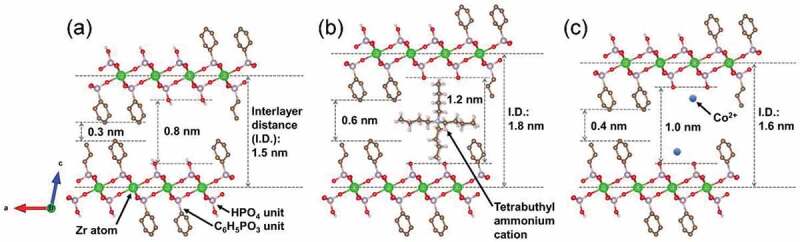
A schematic structure of ZP-Ph-0.5 based on the proposed crystalline structure of zirconium phenylphosphonate by Poojary et al [28]. (a) as prepared sample, (b) samples treated with TBA-solution, and (c) samples treated with TBA-solution followed by treatment with CoCl_2_ solution

The ions of therapeutic inorganic elements, such as cobalt, were introduced into the samples, which were mediated by the temporal insertion of TBA cations. The IDs of the organically modified LZPs increased after immersion in TBA solution, whereas the extent of increase heightened as the quantity of phosphate groups rose in the prepared samples. The increase in ID followed the order of ZP-Ph-0 (increase of 1.0 nm) > ZP-Ph-0.25 (increase of 0.5 nm) > ZP-Ph-0.5 (increase of 0.3 nm) > ZP-Ph-0.75 (increase of 0.1 nm) ˃ ZP-Ph-1.0 (increase of 0.1 nm). Hence, the increase in ID was attributed to the exchange of protons at the phosphate groups with the TBA cations. ZP-Ph-0.25, ZP-Ph-0.5, and ZP-Ph-0.75 showed relatively small yields after treatment with TBA solution, followed by centrifugation. These phenomena correspond to the elimination of exfoliated products during centrifugation. The ZP-Ph-1.0 showed the second-largest yield. This sample contained the smallest molar fraction of the HPO_4_ group (Table 3). The exfoliation process of ZP-Ph-1.0 is considered to be retarded owing to the minimum content of PO-H groups, which act as ion-exchange sites. ZP-Ph-0 had the lowest SSA and the highest yield among the samples. The exfoliation process of ZP-Ph-0 was considered to proceed slowly compared to that of the other samples. An acetonitrile solution containing CoCl_2_ was used to treat organically modified LZP samples. The solvent was chosen as it is aprotic and, hence, suppresses ion exchange between the TBA cation and H^+^. During this treatment, all samples were supplemented with Co^2+^ in excess of their estimated ion-exchange capacities. After immersion in CoCl_2_ solution, ZP-Ph-0.5 showed the maximum content of Co^2+^, accompanied by a decrease in ID. Therefore, it is suggested that the TBA cations at the interlayer of ZP-Ph-0.5 are exchanged with Co^2+^. ZP-Ph-0.25 contained less Co^2+^ than ZP-Ph-0.5, even though the yield of dried samples after treatment with TBA was similar. The peaks attributable to the 002 reflections were negligibly small in the XRD patterns of ZP-Ph-0.25. Hence, the disordered ID in ZP-Ph-0.25 leads to a relatively small uptake of Co^2+^. ZP-Ph-0.75 and ZP-Ph-1.0 showed relatively small uptakes of Co^2+^, with minimum changes in the IDs among the samples. These phenomena were correlated with the relatively small content of phosphate groups in both samples. Figure 11 shows the schematic structure of ZP-Ph-0.5, based on the crystalline structure of zirconium phenylphosphonate proposed by Poojary et al. [28]. The as-prepared ZP-Ph-0.5 had an ID of approximately 1.5 nm, consistent with the proposed structure. The size of the vacancy between the two phenyl or two phosphate groups was approximately 0.3 and 0.8 nm, respectively (Figure 11(a)). After treatment with TBA, the ID increased to 1.8 nm; hence, the distance between the two phosphate groups was estimated to be 1.2 nm. The TBA cation is considered to bind to the phosphate groups because the estimated distance matches the molecular size of a single TBA cation (Figure 11(b)). The ID decreased to 1.6 nm after treatment with CoCl_2_. This decrease in ID was attributed to the exchange of the TBA cation with Co^2+^ (Figure 11(c)). These results demonstrate the possible use of TBA cations as the temporal ID increasing agent with the ability to be easily exchanged with other inorganic cations through a simple immersion process under ambient conditions.

The amounts of Co^2+^, Cu^2+^, and Fe^3+^ introduced into 1 g of ZP-Ph-0.5 were determined to be 0.3, 1.0, and 0.5 mmol·g^−1^, respectively. These values corresponded to 18, 54, and 41% of the theoretical ion-exchange capacity in ZP-Ph-0.5 (3.7 mmol(+)·g^−1^). The rapid dissolution of Co^2+^ and Cu^2+^ was observed within the initial 2 h, followed by gentle sustained release for up to 672 h. The rates of sustained release were 0.1–0.5 µmol·L^−1^·h^−1^ for both ions. Release of Fe^3+^ from the samples was negligible. Therefore, Fe^3+^ was found to be fixed more firmly to the organically modified LZP than Co^2+^ and Cu^2+^.

Co^2+^ release behavior from ZP-Ph-0.5 was also evaluated in SBF for investigation in an environment that mimics human body fluids. Sustained release of Co^2+^ was observed from 2 h to 24 h after immersion. The release rate was 2–37 µmol·L^−1^·h^−1^. The release of Co^2+^ in water was negligible throughout the 24 h experiment. These results suggest that sustained release of Co^2+^ from organically modified LZPs is facilitated by ion exchange with inorganic ions, including Na^+^, in saline or SBF.

The particles of drug delivery materials are often embedded into polymer scaffolds and/or coating films for use in vivo. Here, Co^2+^-incorporated ZP-Ph-0.5 powder was hot-pressed onto one side of the PE substrate, and then immersed in SBF or saline. The mass-to-volume ratio of ZP-Ph-0.5-to-media was identical to that of the immersion test of the powder samples. The Co^2+^ concentrations in the saline and SBF reached 0.125 and 0.062 mmol·L^−1^, respectively, after immersion of the substrates for 24 h. These values correspond to approximately one-third of the accumulated concentrations of Co^2+^ released from the powder sample during the same period.

These concentrations are very close to the effective concentration range of Co^2+^ for stimulation of angiogenesis by vascular endothelial cells. A previous report showed that Co^2+^ in the range of 3–12 ppm (0.051–0.204 mmol·L^−1^) induced the expression of vascular endothelial growth factor in human umbilical vein endothelial cells (HUVECs) cultured on cobalt-containing bioactive glass scaffolds [19]. Cu^2+^ released from Cu-doped calcium silicate ceramics at a concentration of 0.7 μg·mL^−1^ (0.011 mmol·L^−1^) was reported to promote angiogenesis in HUVECs [29]. Here, ZP-Ph-0.5 showed sustained release of Co^2+^ and Cu^2+^ at concentrations within the therapeutic range. Therefore, ZP-Ph-0.5 is a potential material for tissue repair utilizing the sustained release of inorganic ions.

## 5. Conclusion

Layered zirconium phosphate/phosphonate (LZP) with approximately 35 mol% phenyl modification (ZP-Ph-0.5) showed improved incorporation and controlled release of therapeutic inorganic ions. Organically modified derivatives of LZP were successfully prepared with various molar fractions of phenyl groups through reflux processing. When immersed in an acetonitrile solution containing CoCl_2_, the sample prepared with an equimolar phosphoric acid/phenylphosphonic acid mixture (ZP-Ph-0.5) showed the maximum Co^2+^ content among the samples. ZP-Ph-0.5 was also capable of being loaded with Cu^2+^ and Fe^3+^. The sustained release of Co^2+^ and Cu^2+^ was observed up to 672 h after the immersion of ZP-Ph-0.5 in saline solution. The sample also showed controlled release of Co^2+^ in the simulated body fluid (SBF) for 24 h.
